# Supplementary material for: Transcriptome, Ectopic Expression and Genetic Population Analysis Identify Candidate Genes for Fiber Quality Improvement in Cotton
Source: Int J Mol Sci. 2023 May 5;24(9):8293. doi: 10.3390/ijms24098293 (PMC10179096; doi:10.3390/ijms24098293)
Supplement: Supplementary file 1 [file ijms-24-08293-s001.zip › Supplemantary Figures.pdf]

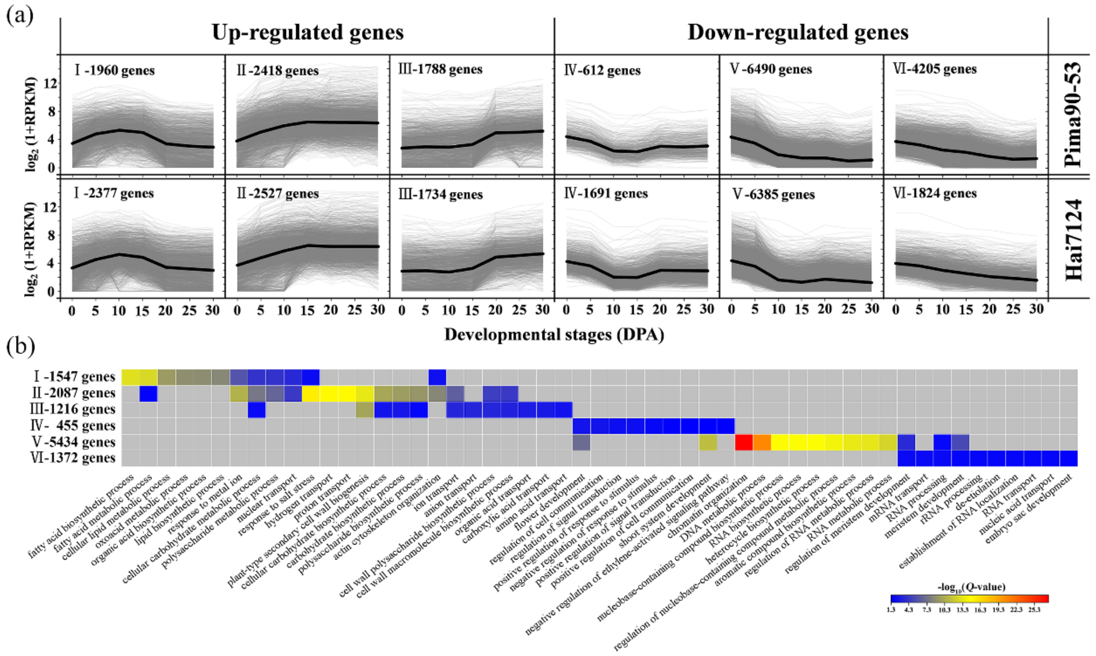

Supplementary Figure S1 Expression patterns and GO enrichment analysis of DEGs. (**a**) Expression patterns of DEGs during cotton fiber development in *G. barbadense* acc. Pima90-53 and Hai7124. DEGs were up-regulated or down-regulated significantly at 5, 10, 15, 20, 25 and 30 DPA as compared with 0 DPA. DEGs were divided into six categories based on expression trends. (**b**) GO enrichment analysis of DEGs in the different categories. The common genes of Pima90-53 and Hai7124 in each category were used for GO enrichment analysis. GO terms with *Q*-value < 0.05 were considered significantly enriched.

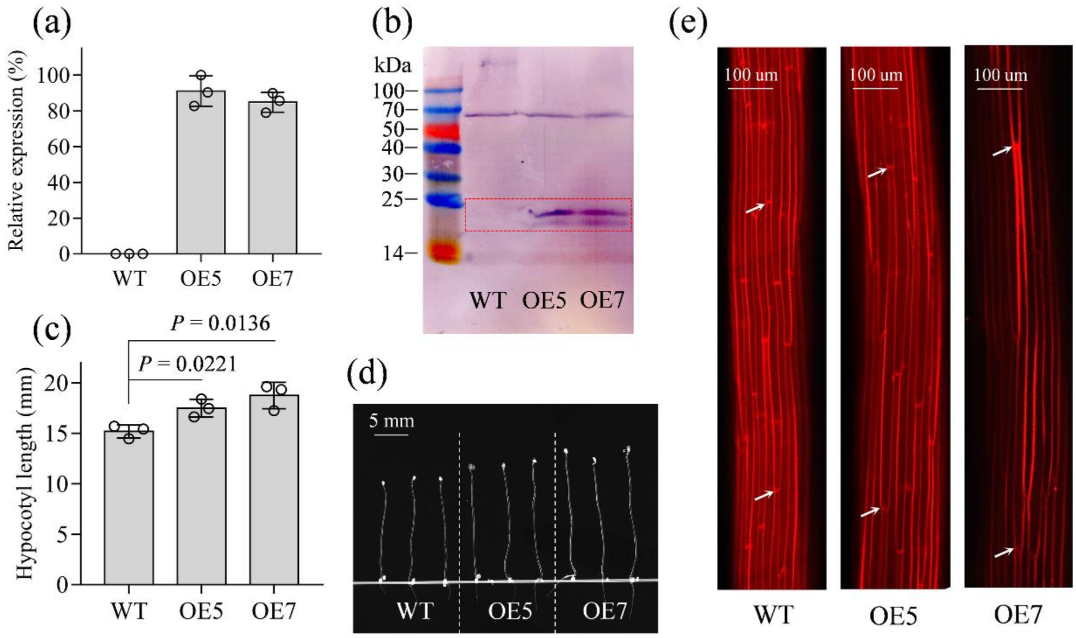

Supplementary Figure S2 Functional analysis of *GbTWS1* in *Arabidopsis*. (**a**) Expression of *GbTWS1* in *GbTWS1*-overexpressed *Arabidopsis* plants, detected by qRT-PCR. (**b**) Western blot of GbTWS1 in *GbTWS1*-overexpressed *Arabidopsis* plants. (**c-d**) *Arabidopsis* plants with overexpression of *GbTWS1*, compared with wild type, showed significantly longer dark-grown hypocotyls. (**e**) Microscopic inspection of hypocotyl epidermal cells. The arrows mark the ends of representative epidermal cells.

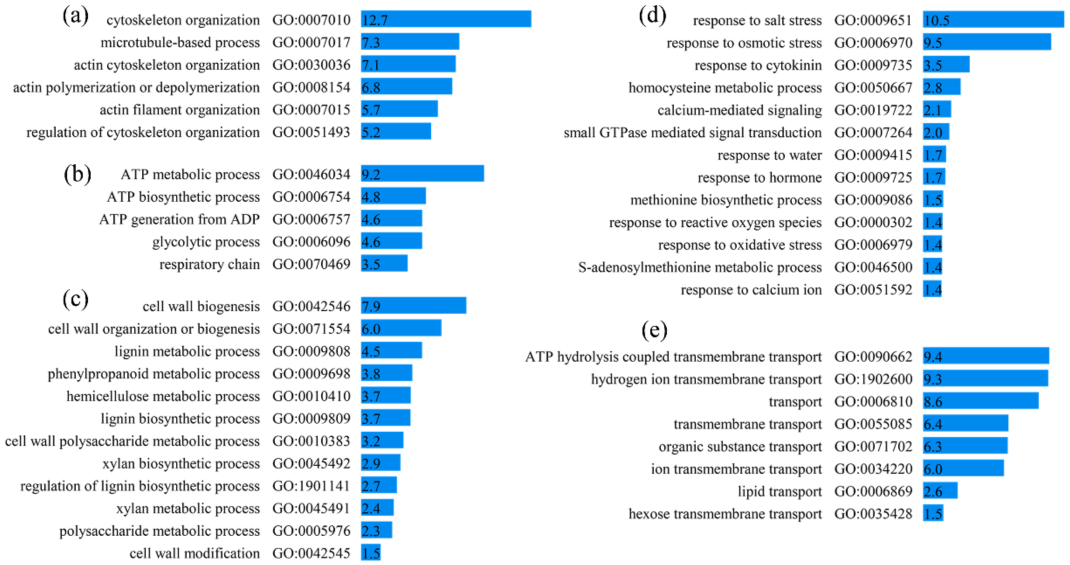

Supplementary Figure S3 GO enrichment analysis of the group I preferentially expressed genes during SCW biosynthesis. The significantly enriched GO terms were mainly classified into five categories: cytoskeleton organization (**a**), ATP metabolism (**b**), cell wall development (**c**), gene expression regulation (**d**), and transport (**e**). GO terms with *Q*-value < 0.05 were considered significantly enriched. The bars show *Q*-value with a $-\log_{10}$ transformation.

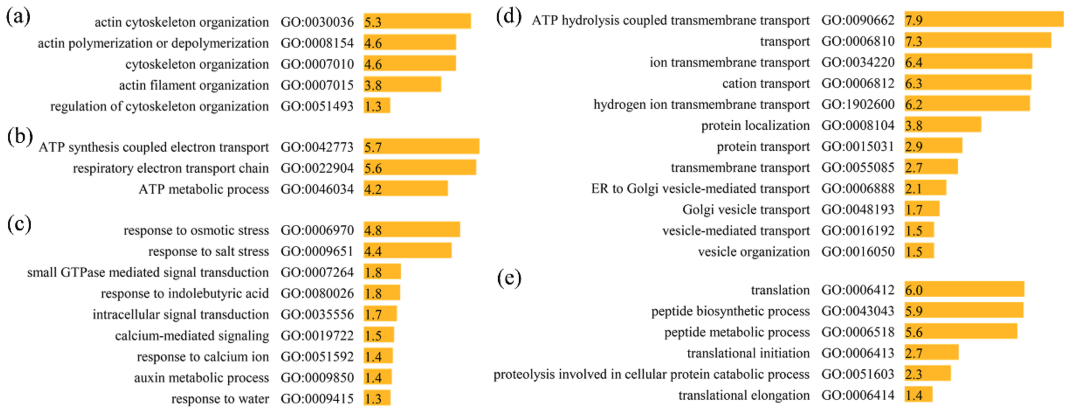

Supplementary Figure S4 GO enrichment analysis of the group II preferentially expressed genes during SCW biosynthesis. The significantly enriched GO terms were mainly classified into five categories: cytoskeleton organization (**a**), ATP metabolism (**b**), gene expression regulation (**c**), transport (**d**), and protein metabolism (**e**).

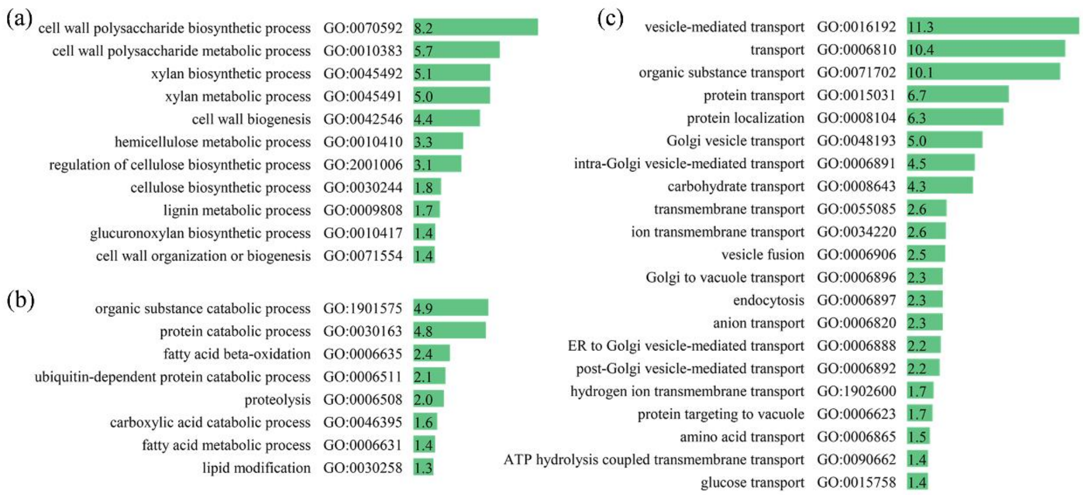

Supplementary Figure S5 GO enrichment analysis of the group III preferentially expressed genes during SCW biosynthesis. The significantly enriched GO terms were mainly classified into three categories: cell wall development (**a**), protein and lipid metabolism (**b**), and transport (**c**).

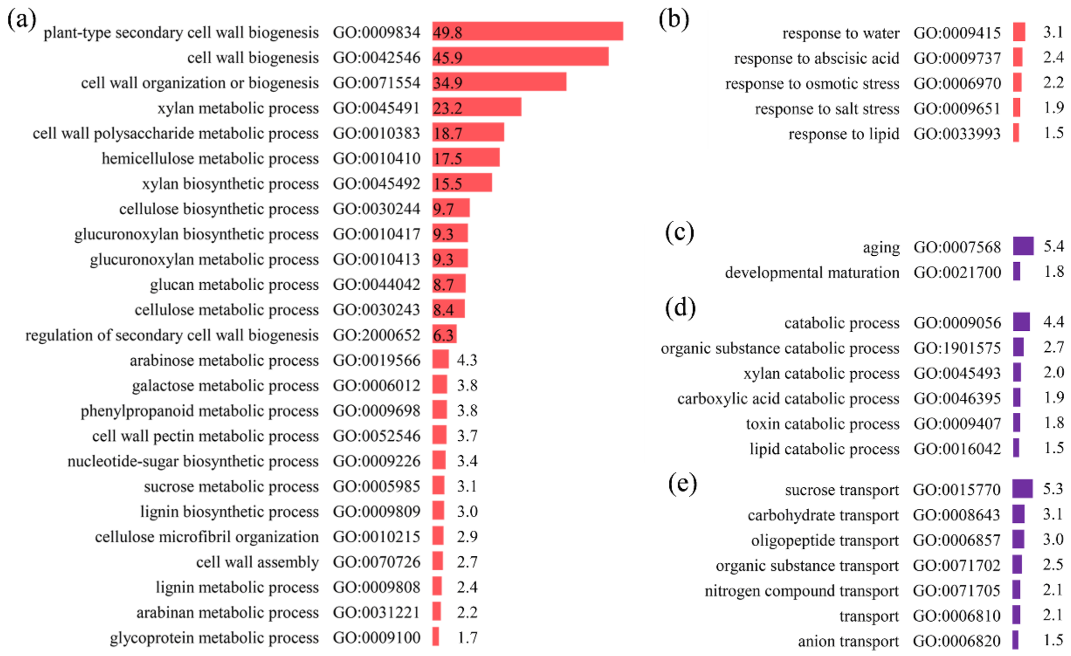

Supplementary Figure S6 GO enrichment analysis of the group IV (**a-b**) and V (**c-e**) preferentially expressed genes during SCW biosynthesis. Diagram exhibits the significantly enriched GO terms related to cell wall development (**a**), gene expression regulation (**b**), maturation (**c**), catabolism (**d**) and transport (**e**).

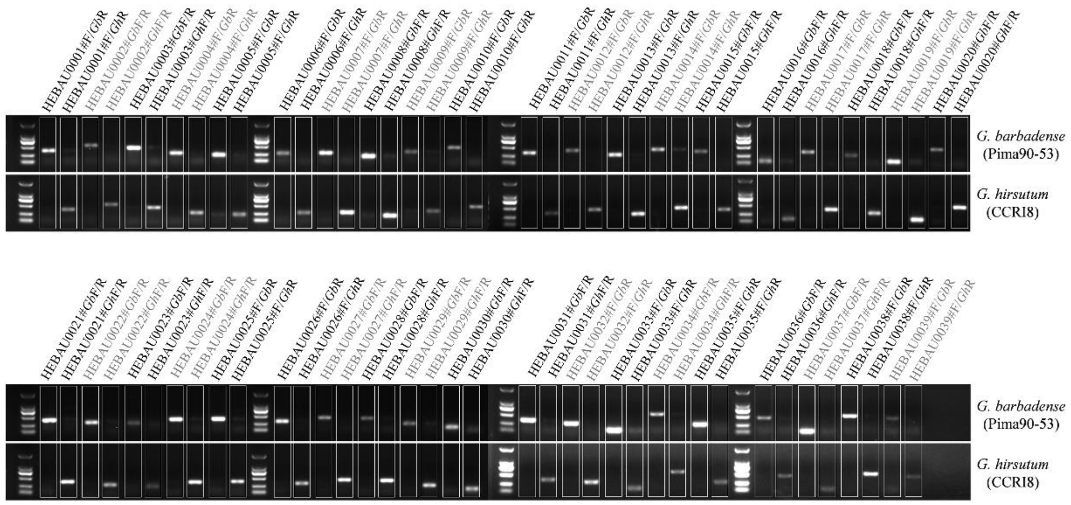

Supplementary Figure S7 Detection of SNP molecular markers in *G. barbadense* and *G. hirsutum*.

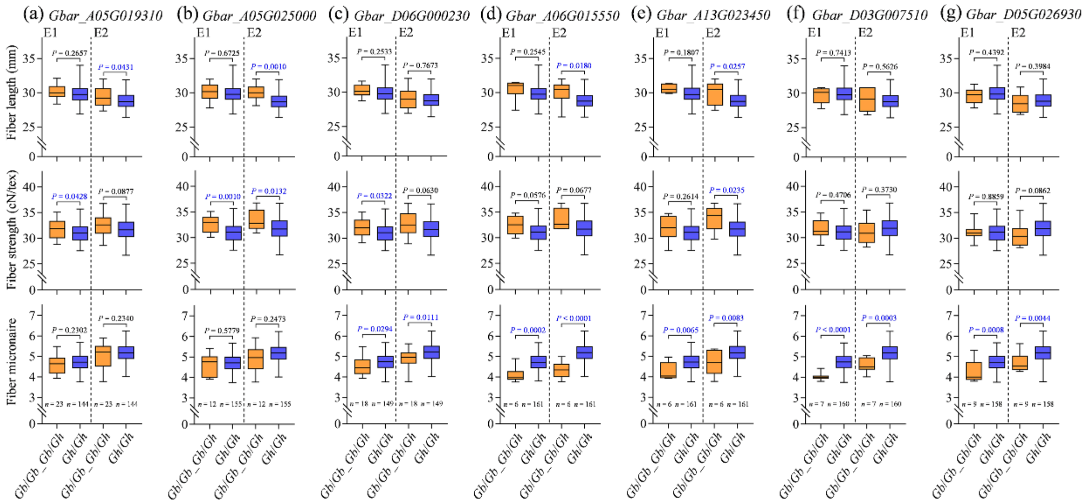

Supplementary Figure S8 The influence on cotton fiber quality with the introgression of *Gbar_A05G019310* (**a**), *Gbar_A05G025000* (**b**), *Gbar_D06G000230* (**c**), *Gbar_A06G015550* (**d**), *Gbar_A13G023450* (**e**), *Gbar_D03G007510* (**f**) and *Gbar_D05G026930* (**g**).
